# Supplementary material for: BECLIN1 is essential for intestinal homeostasis involving autophagy-independent mechanisms through its function in endocytic trafficking
Source: Commun Biol. 2024 Feb 20;7:209. doi: 10.1038/s42003-024-05890-7 (PMC10879175; doi:10.1038/s42003-024-05890-7)
Supplement: Supplementary file 2 — Supplementary Information [file 42003_2024_5890_MOESM2_ESM.pdf]

BECLIN1 is essential for intestinal homeostasis involving autophagy-independent mechanisms through its function in endocytic trafficking (Supplementary information)

Sharon Tran[1,2,#], Juliani Juliani[1,2,3,4,#], Tiffany J. Harris[1,2], Marco Evangelista[1,2], Julian Ratcliffe[5], Sarah L. Ellis[1,2], David Baloyan[1,2], Camilla M. Reehorst[1,2], Rebecca Nightingale[1,2], Ian Y. Luk[1,2], Laura J. Jenkins[1,2], Sonia Ghilas[1,2], Marina H. Yakou[1,2], Chantelle Inguanti[1,2], Chad Johnson[5], Michael Buchert[1,2], James C. Lee[6,7], Peter De Cruz[8,9], Kinga Duszyc[10], Paul A. Gleeson[11], Benjamin T. Kile[12], Lisa A. Mielke[1,2], Alpha S. Yap[10], John M. Mariadason[1,2], W. Douglas Fairlie[1,2,3,4,*], Erinna F. Lee[1,2,3,4,*]

[*]Corresponding authors:
E.F. Lee
Erinna.Lee@latrobe.edu.au

W.D. Fairlie
Doug.Fairlie@onjcri.org.au

#These authors contributed equally to the work.

[1]Olivia Newton-John Cancer Research Institute, Heidelberg, VIC, Australia, [2]School of Cancer Medicine, La Trobe University, Bundoora, VIC, Australia, [3]Department of Biochemistry and Chemistry, School of Agriculture, Biomedicine and Environment, La Trobe University, Bundoora, VIC, Australia, [4]La Trobe Institute for Molecular Science, La Trobe University, Bundoora, VIC, Australia, [5]Bioimaging Platform, La Trobe University, Bundoora, VIC, Australia, [6]Genetic Mechanisms of Disease Laboratory, the Francis Crick Institute, London, United Kingdom, [7]Institute of Liver and Digestive Health, Royal Free Hospital, University College London, London, United Kingdom, [8]Department of Gastroenterology, Austin Health, Melbourne, VIC, Australia, [9]Department of Medicine, Austin Academic Centre, The University of Melbourne, Melbourne, VIC, Australia, [10]Institute for Molecular Bioscience, The University of Queensland, St. Lucia, Brisbane, QLD, Australia, [11]Department of Biochemistry and Pharmacology and Bio21 Molecular Science and Biotechnology Institute, The University of Melbourne, Melbourne, VIC, Australia, [12]Garvan Institute of Medical Research, Darlinghurst, NSW, Australia.

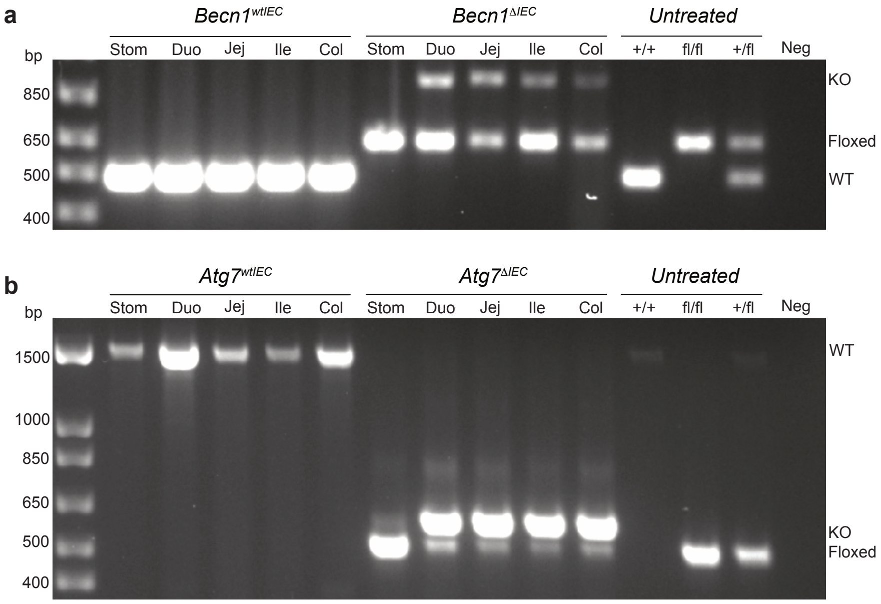

Supplementary Fig. 1 PCR-based genotyping following tamoxifen-induced deletion of *Becn1* and *Atg7* in adult *Becn1*$^{fl/fl}$;*Vil1-CreERT2*$^{Cre/+}$- and *Atg7*$^{fl/fl}$;*Vil1-CreERT2*$^{Cre/+}$ -derived intestinal epithelial cells. Stom: stomach. Duo: duodenum. Jej: jejunum. Ile: ileum. Col: colon. KO: knock-out. WT: wild-type

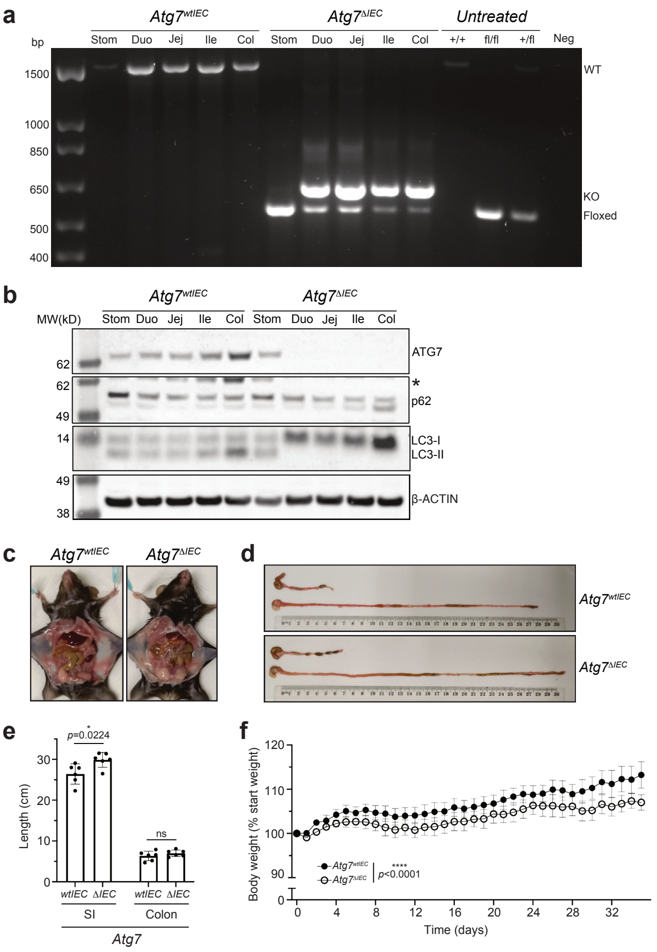

Supplementary Fig. 2 Intestinal epithelium-specific loss of ATG7 in adult mice leads to slightly longer small intestinal lengths and decreased body weight gain which develop over one month. **a** PCR-based genotyping and **b** Western blotting demonstrated successful deletion of ATG7 in intestinal epithelial cells. Representative images of **c** abdominal necropsy and **d** intestinal tracts demonstrating slightly increased small intestinal, but not colon, lengths. However, there was no evidence of severe loss of intestinal homeostasis seen in the absence of BECLIN1 over a significantly shorter time frame. **e** The absence of ATG7 over this extended period of time leads to a decrease in body weight gain. For all data represented, at least n=6 biologically independent mice of each genotype were used. Data represent n=3 independent experiments unless otherwise indicated. All graphs show the mean ± S.E.M. Significance was determined by Wilcoxon's t-test. Stom: stomach. Duo: duodenum. Jej: jejunum. Ile: ileum. Col: colon. KO: knock-out. WT: wild-type. *: non-specific band.

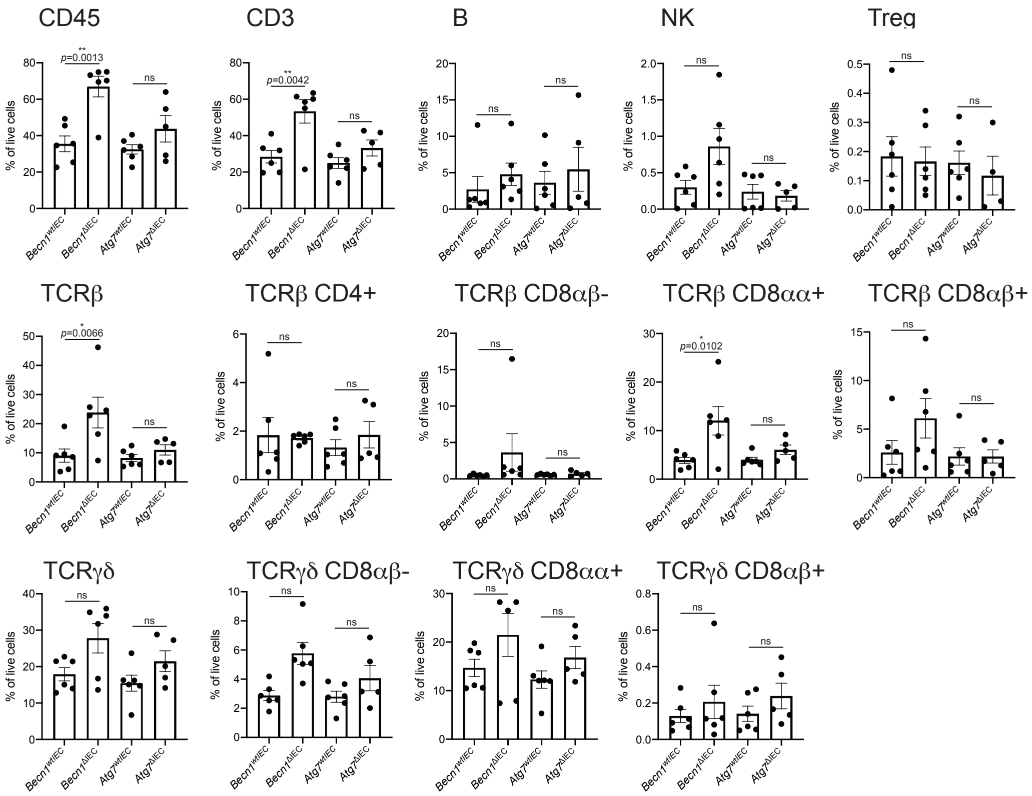

Supplementary Fig. 3 Immunophenotyping of the intraepithelial lymphocytes in the small intestines. Graphs show the frequency of each indicated immune cell subtype within total live cells. Data represent mean ± S.E.M and significance determined by ordinary one-way ANOVA. Data is representative of at least n=4 biologically independent mice of each genotype from n=2 independent experiments.

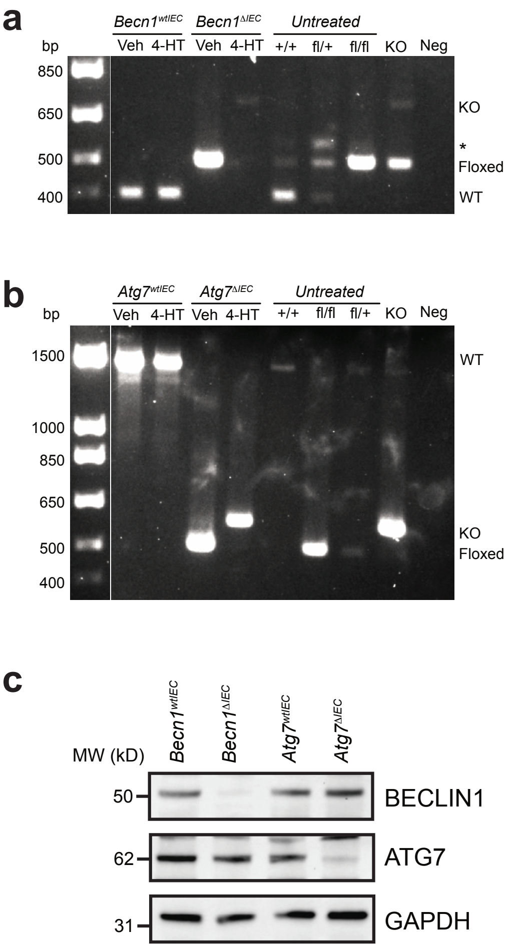

Supplementary Fig. 4 Tamoxifen-induced deletion of *Becn1* and *Atg7* in *Becn1*$^{fl/fl}$;*Vil1-CreERT2*$^{Cre/+}$- and *Atg7*$^{fl/fl}$;*Vil1-CreERT2*$^{Cre/+}$ -derived intestinal organoids as detected by a PCR genotyping and b Western blotting. *: non-specific band.

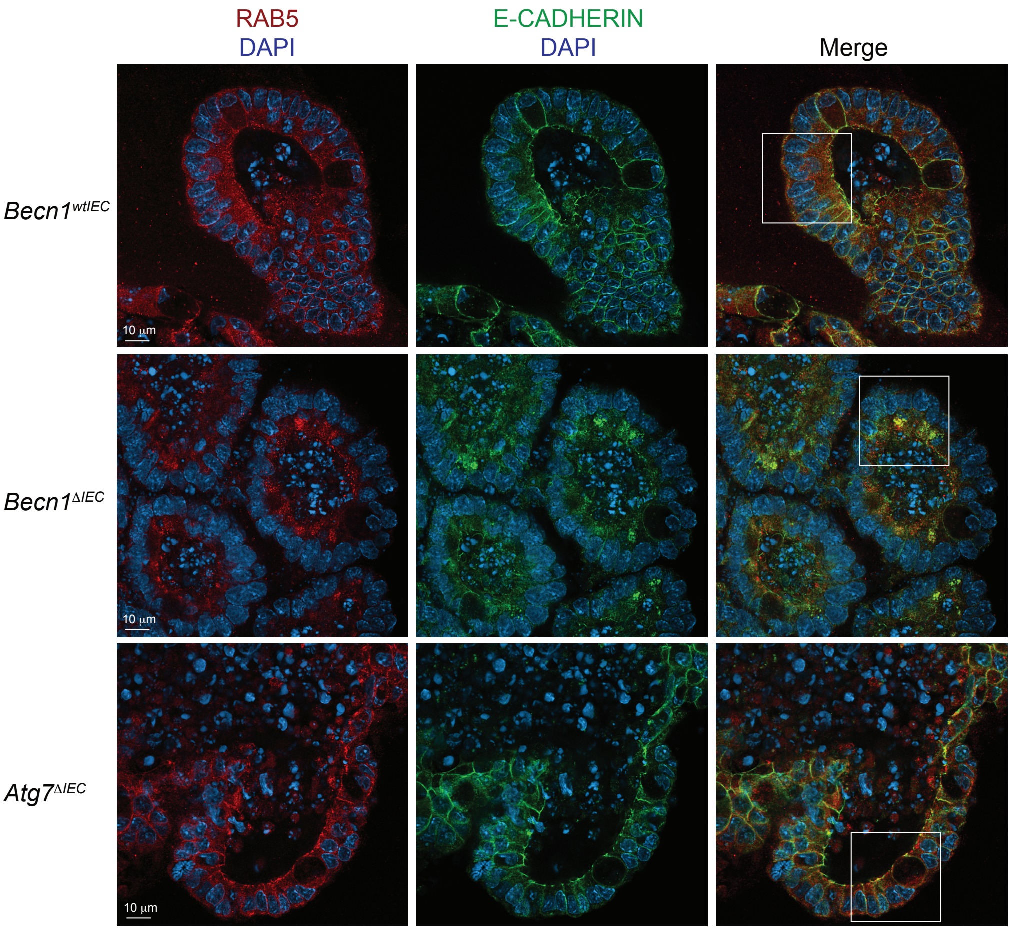

Supplementary Fig. 5 Immunofluorescence staining of E-CADHERIN and RAB5^{+ve} early endosomes. Immunofluorescence stained wild-type, *Becn1$^{\Delta IEC}$* and *Atg7$^{\Delta IEC}$* intestinal organoids were assessed by confocal microscopy. Images are presented at 68x zoom. The white box in the merged images refer to the areas represented in Fig. 5a. Data are representative of at least n=3 independent experiments with at least n=3 different organoids from each experiment.

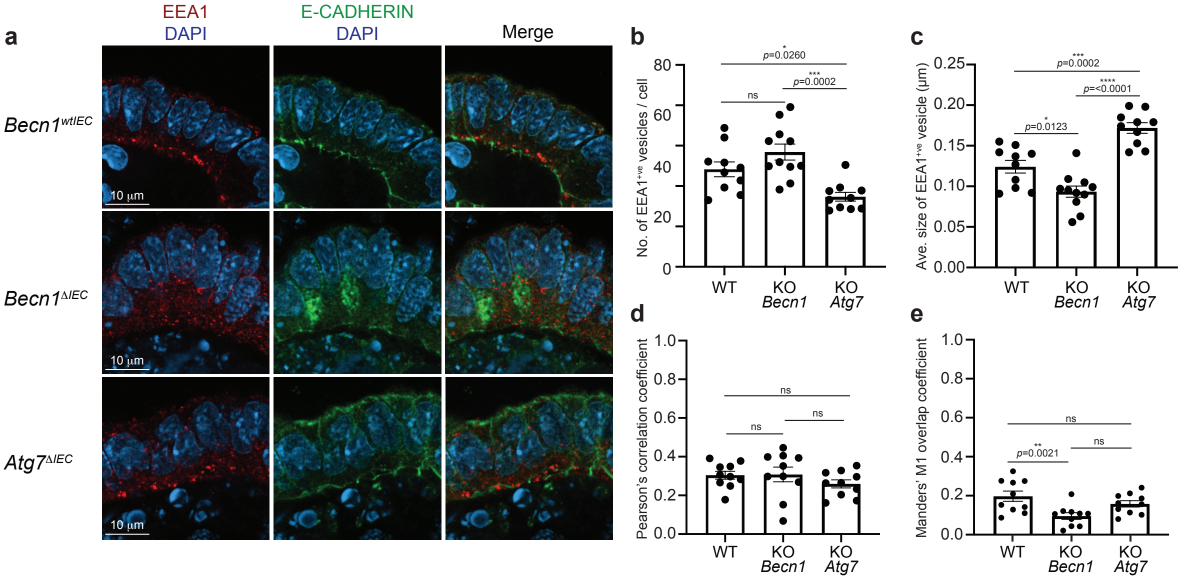

Supplementary Fig. 6 Loss of BECLIN1 leads to aberrant EEA1+ve recruitment to early endosomes with no change in colocalisation observed between E-CADHERIN and EEA1^{+ve} vesicles. a The absence of BECLIN1, but not ATG7, leads to loss of apical membrane staining of EEA1 as detected by whole-mount immunofluorescent staining of wild-type, *Becn1^{ΔIEC}* and *Atg7^{ΔIEC}* intestinal organoids. **b** Loss of BECLIN1 did not cause changes to the size of EEA1^{+ve} vesicles, though loss of ATG7 did. **c** There were significant changes observed in the average size of EEA1^{+ve} vesicles when either BECLIN1 or ATG7 was absent. **d, e** The absence of BECLIN1 did not increase the amount of E-CADHERIN colocalisation with EEA1^{+ve} early endosomes unlike that seen with RAB5^{+ve} early endosomes suggesting a failure to recruit EEA1 to the compartment. Data are representative of at least n=3 different slices per organoid and of at least n=3 different organoids from three independent experiments. Graphs indicate the mean ± S.E.M. Significance was determined by ordinary one-way ANOVA for endosomal numbers, size and the Pearson's correlation coefficient and by two-way ANOVA for the Mander's M1 overlap coefficient.

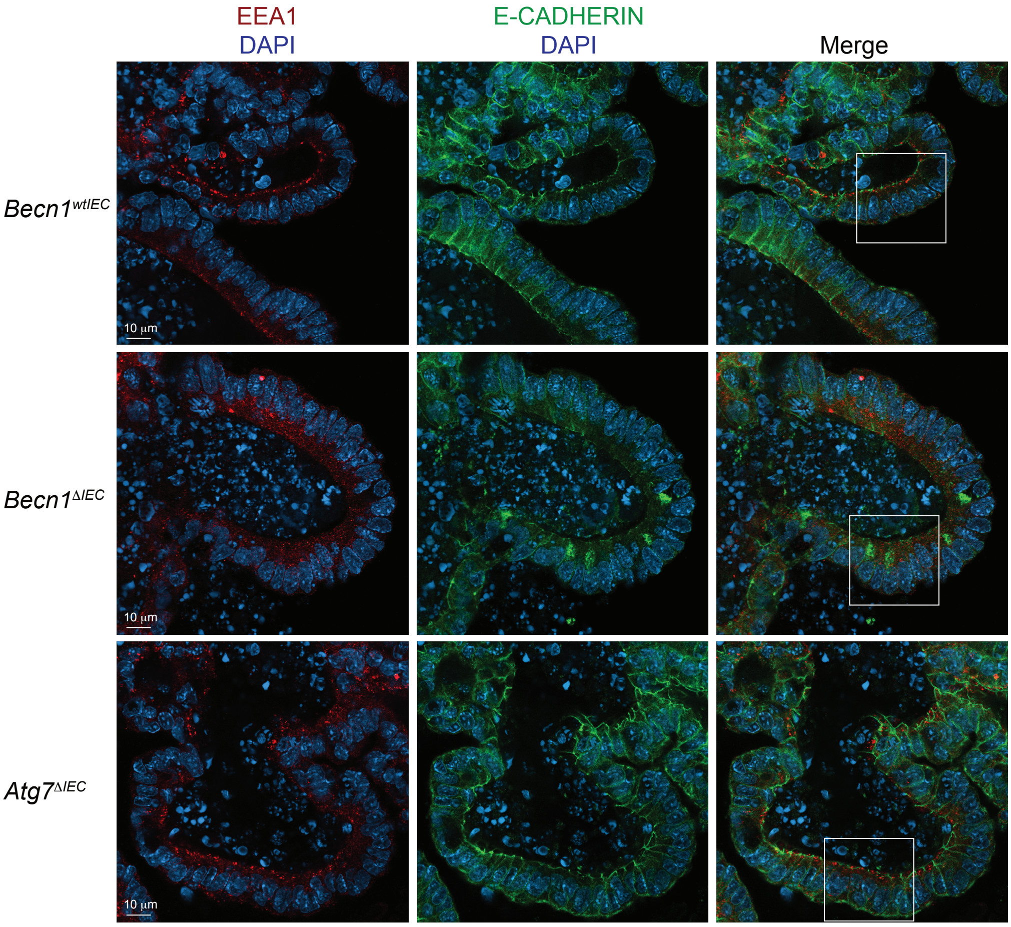

Supplementary Fig. 7 Immunofluorescence staining of E-CADHERIN and EEA1^{+ve} early endosomes. Immunofluorescence stained wild-type, *Becn1$^{\Delta IEC}$* and *Atg7$^{\Delta IEC}$* intestinal organoids were assessed by confocal microscopy. Images are presented at 68x zoom. The white box in the merged images refer to the areas represented in Supp Fig. 6. Data are representative of at least n=3 independent experiments with at least n=3 different organoids from each experiment.

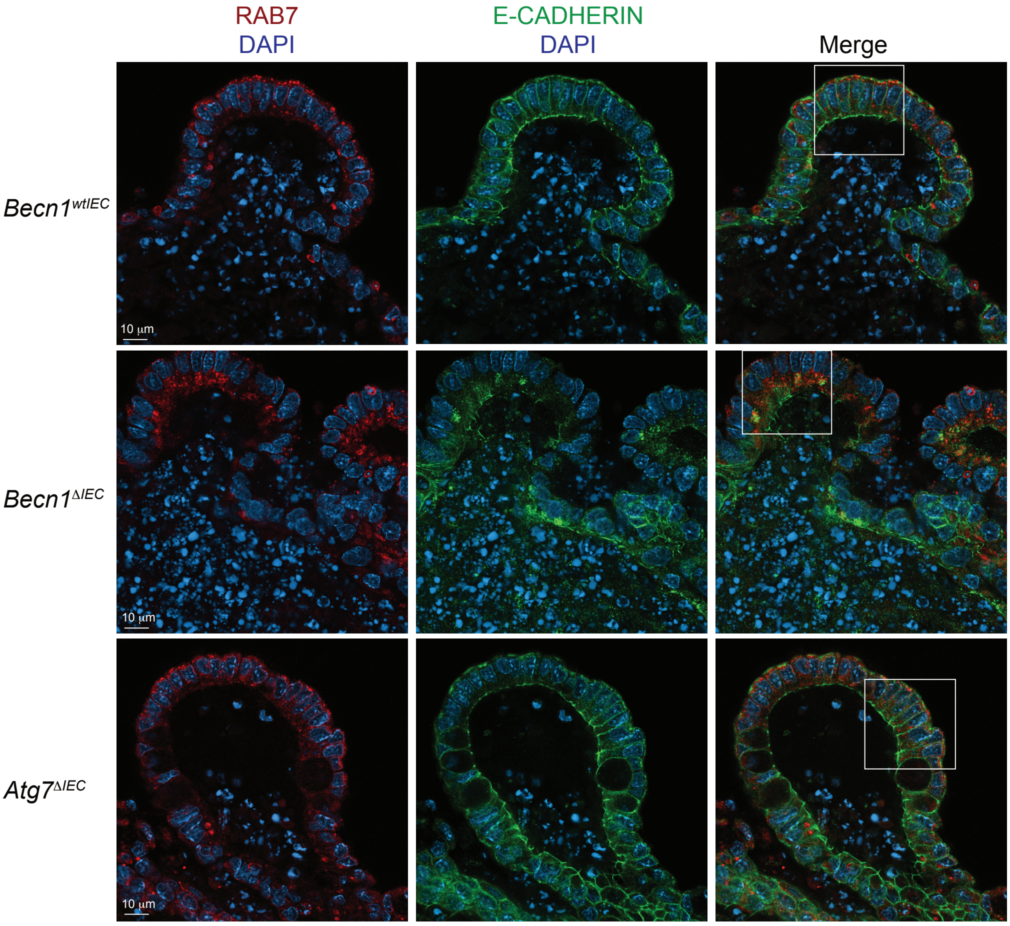

Supplementary Fig. 8 Immunofluorescence staining of E-CADHERIN and RAB7^{+ve} late endosomes. Immunofluorescence stained wild-type, *Becn1*$^{\Delta IEC}$ and *Atg7*$^{\Delta IEC}$ intestinal organoids were assessed by confocal microscopy. Images are presented at 68x zoom. The white box in the merged images refer to the areas represented in Fig. 5f. Data are representative of at least n=3 independent experiments with at least n=3 different organoids from each experiment.

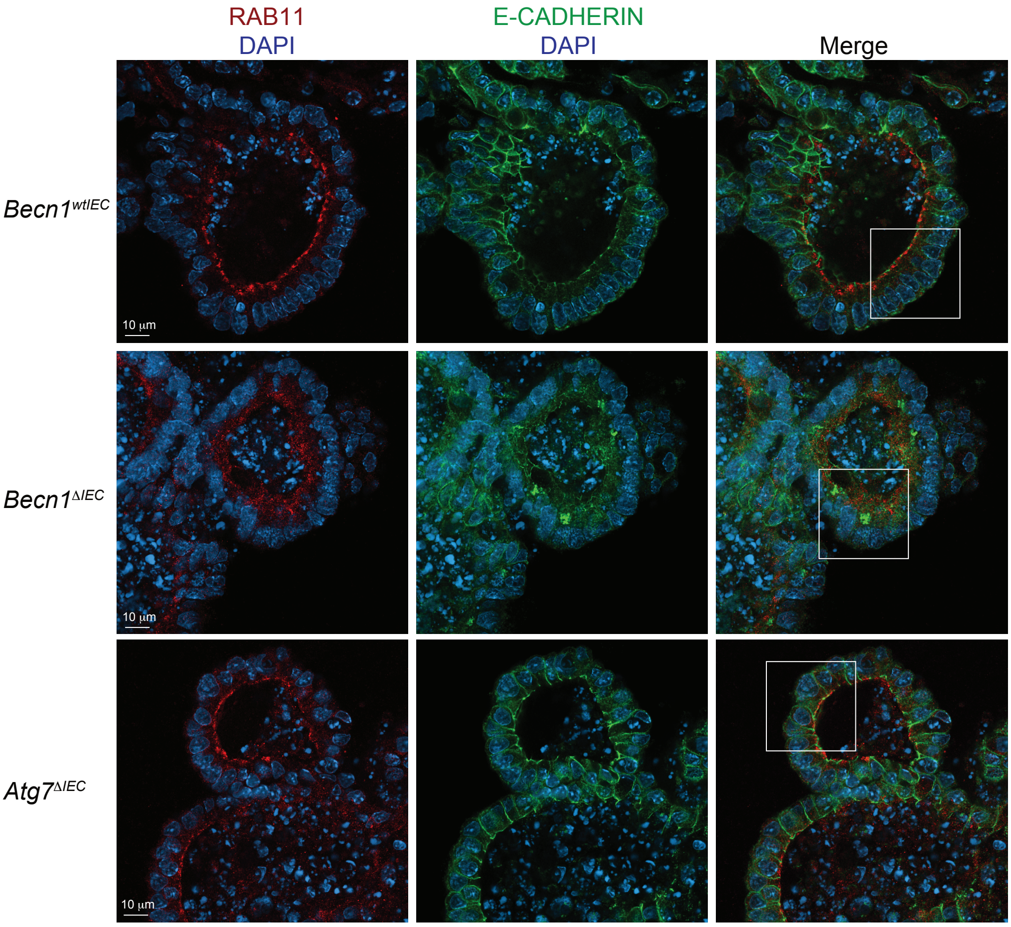

Supplementary Fig. 9 Immunofluorescence staining of E-CADHERIN and RAB11^{+ve} recycling endosomes. Immunofluorescence stained wild-type, *Becn1$^{\Delta IEC}$* and *Atg7$^{\Delta IEC}$* intestinal organoids were assessed by confocal microscopy. Images are presented at 68x zoom. The white box in the merged images refer to the areas represented in Fig. 5k. Data are representative of at least n=3 independent experiments with at least n=3 different organoids from each experiment.

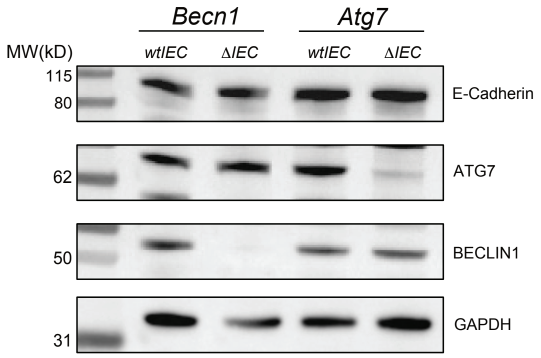

Supplementary Fig. 10 BECLIN1 does not regulate total cellular levels, of E-CADHERIN in intestinal epithelial cells. Whilst the cellular localisation of E-CADHERIN is altered when BECLIN1 is absent, the levels of E-CADHERIN remain the same. Data are representative of at least n=3 independent experiments.

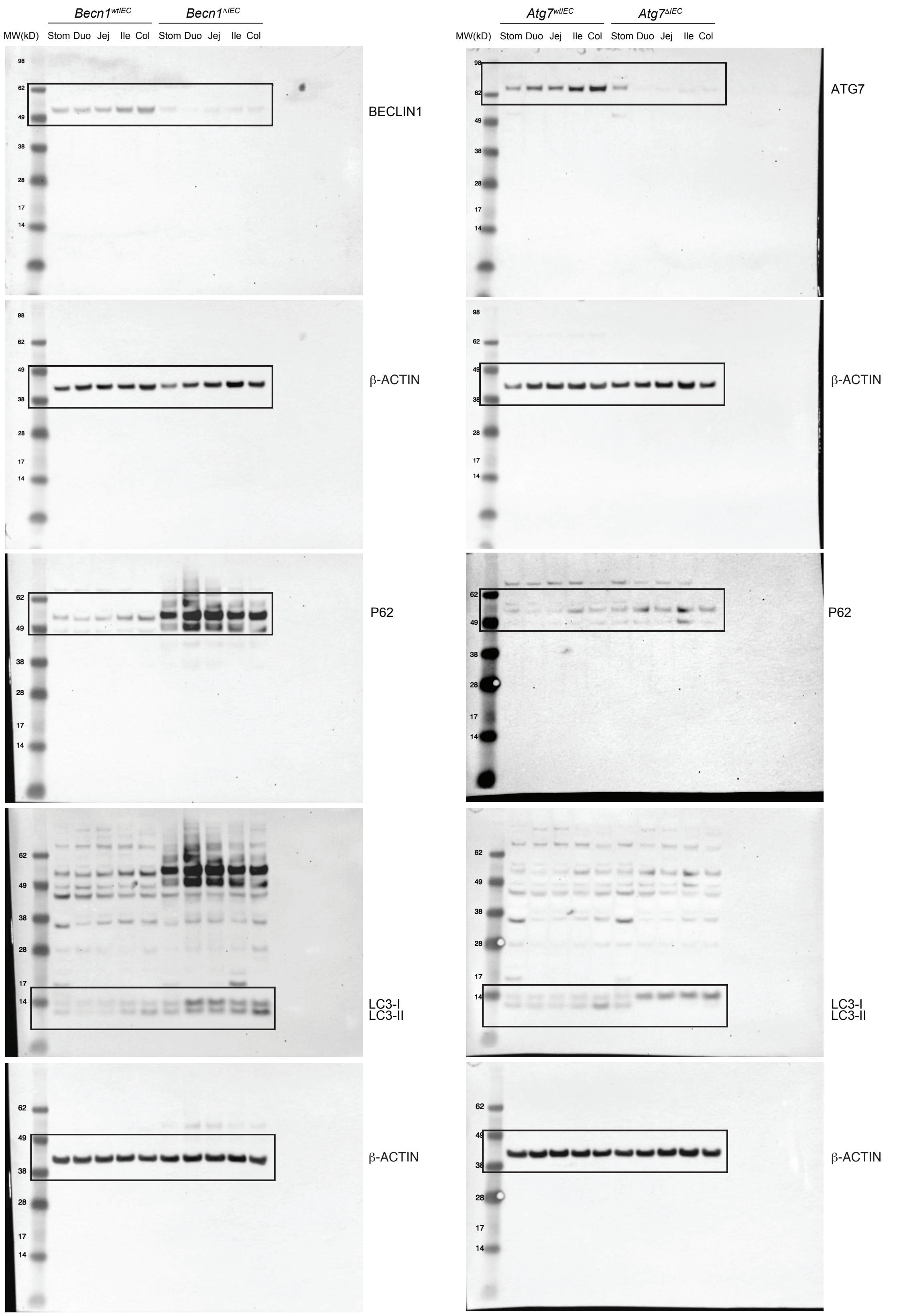

Supplementary Fig. 11 Original immunoblots from Fig. 1b. Intestinal epithelial cell lysates from *Becn1*^{wtIEC}, *Becn1*$^{\Delta IEC}$, *Atg7*^{wtIEC} and *Atg7*$^{\Delta IEC}$ mice were probed for BECLIN1, ATG7,

P62, LC3B and β-ACTIN. Identified rectangles represent the cropped image in the corresponding figure; each Western blot is representative of n=3 independent experiments.

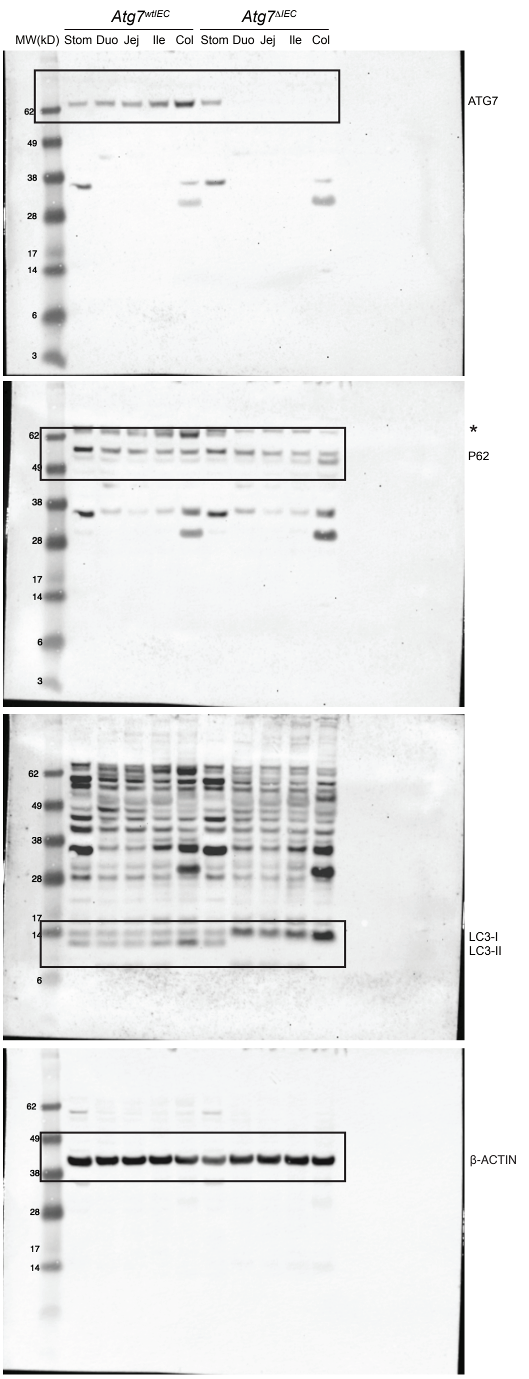

Supplementary Fig. 12 Original immunoblots from Supplementary Fig. 2b. Intestinal epithelial cell lysates from *Atg7*^{wtIEC} and *Atg7*$^{\Delta IEC}$ mice were probed for ATG7, P62, LC3B and β-ACTIN. Identified rectangles represent the cropped image in the corresponding figure; each Western blot is representative of n=3 independent experiments.

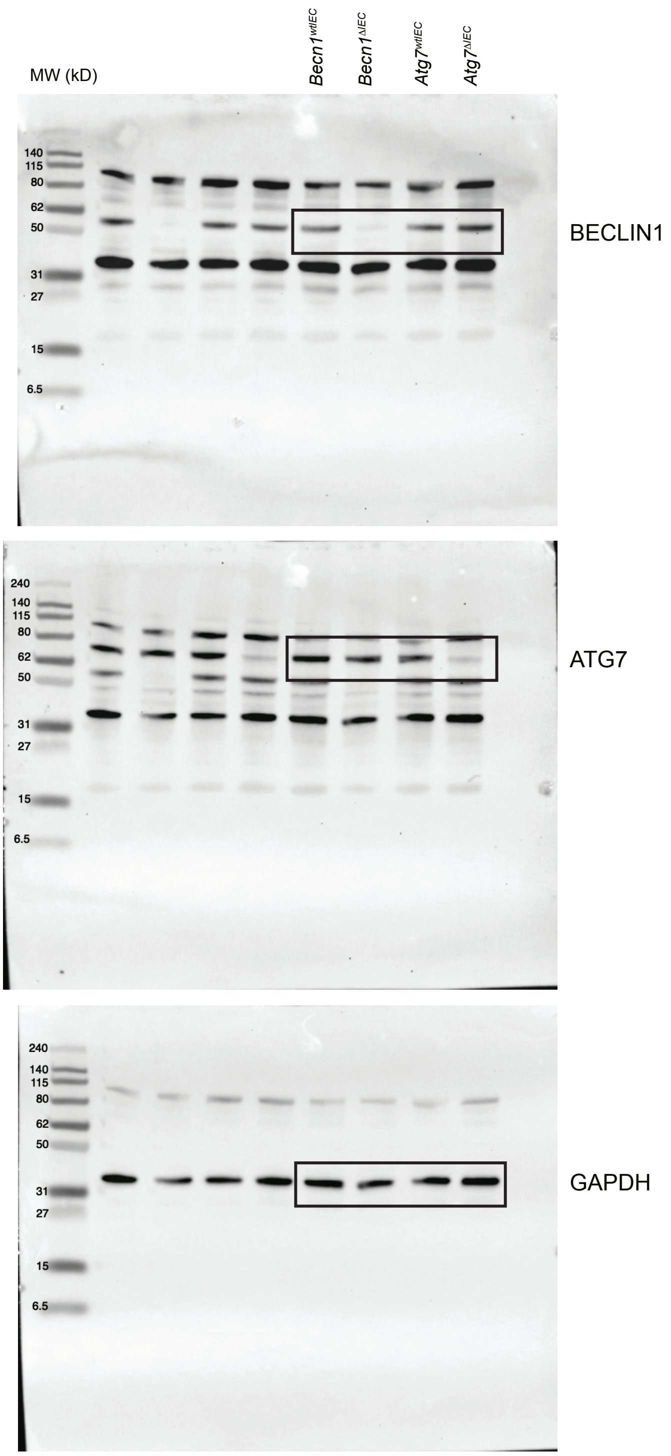

Supplementary Fig. 13 Original immunoblots from Supplementary Fig. 4c. Lysates from *Becn1*^{wtIEC}, *Becn1*^{ΔIEC}, *Atg7*^{wtIEC} and *Atg7*^{ΔIEC} organoids were probed for BECLIN1, ATG7 and GAPDH. Identified rectangles represent the cropped image in the corresponding figure; each Western blot is representative of n=3 independent experiments.

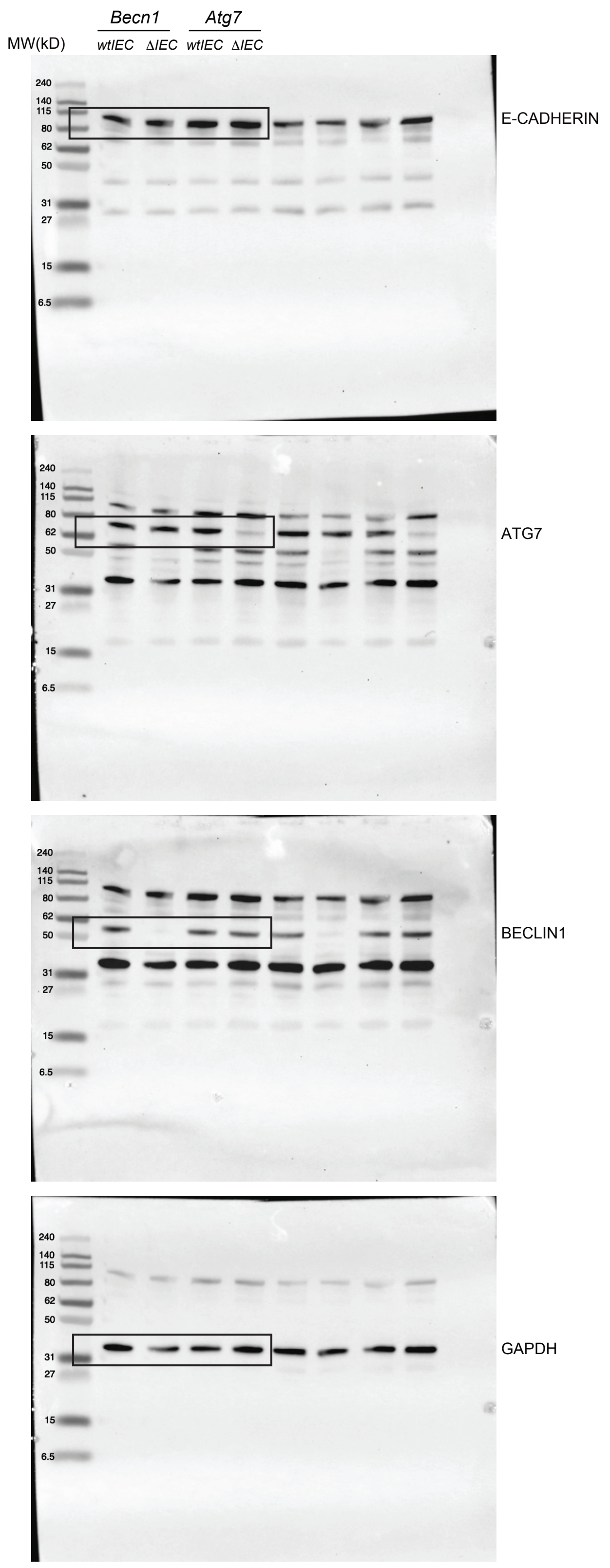

Supplementary Fig. 14 Original immunoblots from Supplementary Fig. 10. Lysates from *Becn1*^{wtIEC}, *Becn1*$^{\Delta IEC}$, *Atg7*^{wtIEC} and *Atg7*$^{\Delta IEC}$ organoids were probed for E-CADHERIN, BECLIN1, ATG7 and GAPDH. Identified rectangles represent the cropped image in the corresponding figure; each Western blot is representative of n=3 independent experiments.

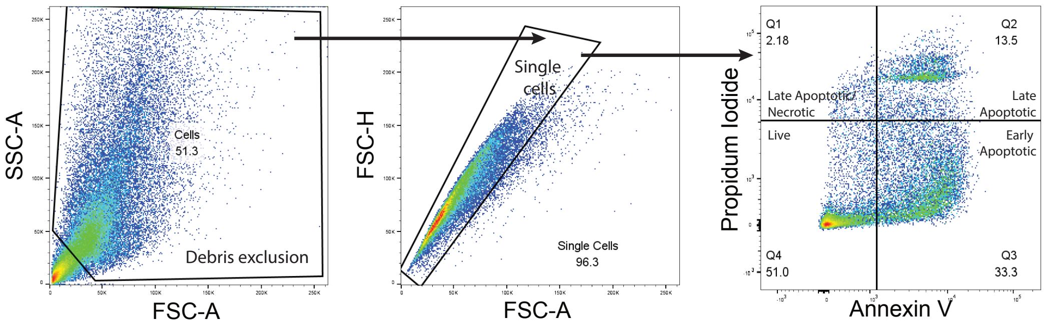

Supplementary Fig. 15 Additional flow cytometry data for the detection of apoptotic epithelial cells from intestinal organoids by propidium iodide (PI) / Annexin V staining. Gating strategy using in Fig. 4d.

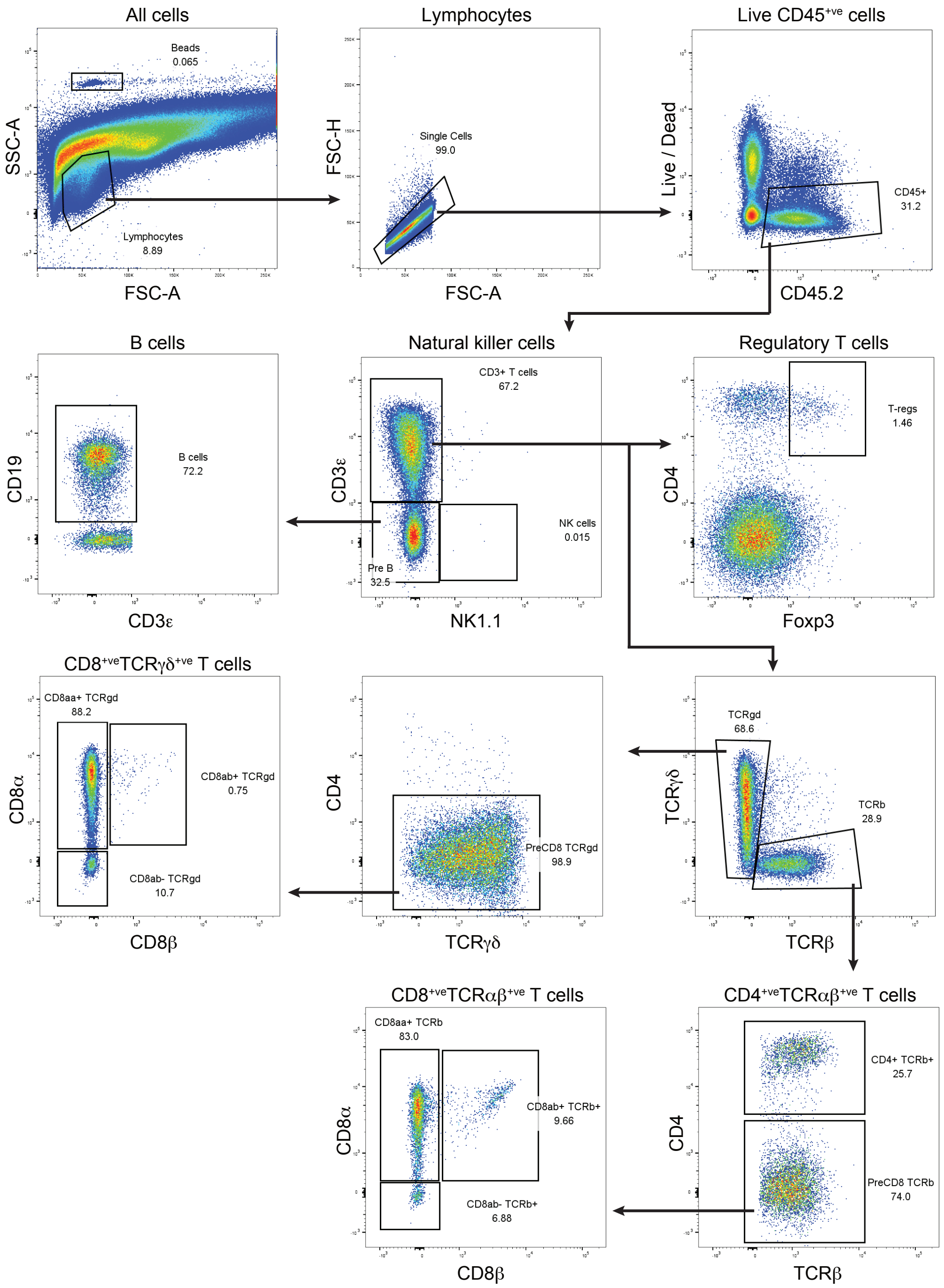

Supplementary Fig. 16 Additional flow cytometry data for immunophenotyping of intraepithelial lymphocytes in the small intestines. Gating strategy using in Supplementary Fig. 3.
