# Supplementary material for: BECLIN1 is essential for intestinal homeostasis involving autophagy-independent mechanisms through its function in endocytic trafficking
Source: Commun Biol. 2024 Feb 20;7:209. doi: 10.1038/s42003-024-05890-7 (PMC10879175; doi:10.1038/s42003-024-05890-7)
Supplement: Supplementary file 3 — Description of additional supplementary files [file 42003_2024_5890_MOESM3_ESM.docx]

Description of Additional Supplementary Files

**File name:** Supplementary Data 1

**Description:** The numerical source data behind all graphs in the paper.
