# Supplementary material for: BECLIN1 is essential for intestinal homeostasis involving autophagy-independent mechanisms through its function in endocytic trafficking
Source: Commun Biol. 2024 Feb 20;7:209. doi: 10.1038/s42003-024-05890-7 (PMC10879175; doi:10.1038/s42003-024-05890-7)
Supplement: Supplementary file 5 — Reporting Summary [file 42003_2024_5890_MOESM5_ESM.pdf]

nature portfolio

Corresponding author(s): Erinna F. Lee
W. Douglas Fairlie

Last updated by author(s): Nov 6, 2023

Reporting Summary

Nature Portfolio wishes to improve the reproducibility of the work that we publish. This form provides structure for consistency and transparency in reporting. For further information on Nature Portfolio policies, see our Editorial Policies and the Editorial Policy Checklist.

Statistics

For all statistical analyses, confirm that the following items are present in the figure legend, table legend, main text, or Methods section.

n/a	Confirmed	
☐	☒	The exact sample size (*n*) for each experimental group/condition, given as a discrete number and unit of measurement
☐	☒	A statement on whether measurements were taken from distinct samples or whether the same sample was measured repeatedly
☐	☒	The statistical test(s) used AND whether they are one- or two-sided<br>*Only common tests should be described solely by name; describe more complex techniques in the Methods section.*
☒	☐	A description of all covariates tested
☒	☐	A description of any assumptions or corrections, such as tests of normality and adjustment for multiple comparisons
☐	☒	A full description of the statistical parameters including central tendency (e.g. means) or other basic estimates (e.g. regression coefficient) AND variation (e.g. standard deviation) or associated estimates of uncertainty (e.g. confidence intervals)
☐	☒	For null hypothesis testing, the test statistic (e.g. *F*, *t*, *r*) with confidence intervals, effect sizes, degrees of freedom and *P* value noted<br>*Give P values as exact values whenever suitable.*
☒	☐	For Bayesian analysis, information on the choice of priors and Markov chain Monte Carlo settings
☒	☐	For hierarchical and complex designs, identification of the appropriate level for tests and full reporting of outcomes
☒	☐	Estimates of effect sizes (e.g. Cohen's *d*, Pearson's *r*), indicating how they were calculated

Our web collection on statistics for biologists contains articles on many of the points above.

Software and code

Policy information about availability of computer code

Data collection	ChemiDoc Imaging System (Bio-Rad); EnVision+System (DAKO); BD FACSCanto II and BD FACSymphonyTM A3 Cell Analyzer (Becton Dickinson and Company, BD Biosciences), Joel JEM-2100 transmission electron microscope at 80kV with Gatan digital camera, Zeiss LSM 980 with Airyscan 2 confocal microscope
Data analysis	FLOWJO v10.5.3 (Becton Dickinson and Company, BD Biosciences); PRISM v8.2.0; Image Lab (Bio-Rad); Zen Blue (Zeiss), ImageJ software (Fiji)

For manuscripts utilizing custom algorithms or software that are central to the research but not yet described in published literature, software must be made available to editors and reviewers. We strongly encourage code deposition in a community repository (e.g. GitHub). See the Nature Portfolio guidelines for submitting code & software for further information.

Data

Policy information about availability of data

All manuscripts must include a data availability statement. This statement should provide the following information, where applicable:

- Accession codes, unique identifiers, or web links for publicly available datasets
- A description of any restrictions on data availability
- For clinical datasets or third party data, please ensure that the statement adheres to our policy

Correspondence and requests for materials should be addressed to W. Douglas Fairlie or Erinna F. Lee.

Research involving human participants, their data, or biological material

Policy information about studies with human participants or human data. See also policy information about sex, gender (identity/presentation), and sexual orientation and race, ethnicity and racism.

Reporting on sex and gender	N/A
Reporting on race, ethnicity, or other socially relevant groupings	N/A
Population characteristics	N/A
Recruitment	N/A
Ethics oversight	N/A

Note that full information on the approval of the study protocol must also be provided in the manuscript.

Field-specific reporting

Please select the one below that is the best fit for your research. If you are not sure, read the appropriate sections before making your selection.

☒ Life sciences ☐ Behavioural & social sciences ☐ Ecological, evolutionary & environmental sciences

For a reference copy of the document with all sections, see nature.com/documents/nr-reporting-summary-flat.pdf

Life sciences study design

All studies must disclose on these points even when the disclosure is negative.

Sample size	For all animal studies, at least 4 to 8 biological replicates from 3 independent experiments were conducted. Whilst no sample-size calculation was performed, the sample sizes chosen are sufficient as the phenotype is 100% penetrant and as such enabled statistically significant data to be generated. For cell-based studies, at least 2-3 independent experiments were performed with at least duplicate samples in each replicate.
Data exclusions	No data was excluded (except in rare cases where there were equipment failures during data acquisition such as sample blocking on FACS).
Replication	All attempts at replication were successful.
Randomization	Samples were allocated based on genotypes and time points.
Blinding	Investigators were blinded to the genotype of the animals being processed and analysed and only assigned animal numbers were used.

Reporting for specific materials, systems and methods

We require information from authors about some types of materials, experimental systems and methods used in many studies. Here, indicate whether each material, system or method listed is relevant to your study. If you are not sure if a list item applies to your research, read the appropriate section before selecting a response.

Materials & experimental systems

n/a	Involved in the study	
☐	☒	Antibodies
☒	☐	Eukaryotic cell lines
☒	☐	Palaeontology and archaeology
☐	☒	Animals and other organisms
☒	☐	Clinical data
☒	☐	Dual use research of concern
☒	☐	Plants

Methods

n/a	Involved in the study	
☒	☐	ChIP-seq
☐	☒	Flow cytometry
☒	☐	MRI-based neuroimaging

Antibodies

Antibodies used	Beclin1, 1:500 (CST, 3495); ATG7, 1:500 (Sigma-Aldrich, A2856), p62/SQSTM1, 1:500 (CST, 5114); LC3B, 1:500 (Novus Biologicals, NB100-2220); [illegible]-Actin, 1:5000 (Sigma-Aldrich, A2228); GAPDH, 1:5000 (Invitrogen, MA5-15738); Donkey anti-Rabbit IgG, 1:10,000 (GE

	Healthcare, NA943V), Goat anti-Mouse IgG, 1:10,000 (Sigma-Aldrich, A0168); Cleaved caspase-3, 1:200 (CST, 9661); Ki-67, 1:150 (Invitrogen, MA5-14520); Lysozyme, 1:300 (Thermo Scientific, RB-372-A1); Chromogranin A, 1:500 (Abcam, ab85554); DCAMKL1, 1:400 (Abcam, ab31704);<br>E-cadherin, 1:500 (Invitrogen, 13-1900) and 1:600 (CST 3195); goat anti-Rat IgG (H+L), 1:500 (Invitrogen, A-11077); Live/Dead marker (1:500, BD Biosciences, 566332), anti-CD45.2 (1:200, BD Biosciences, 741957), CD3ε (1:200, BD Biosciences, 564378), CD4 (1:1000, BD Biosciences, 741461), CD8α (1:1000, BD Biosciences, 562283), CD8β (1:1000, BD Biosciences, 741127), TCRβ (1:300, ThermoFisher Scientific, 47-5961-82), TCRγδ (1:300, BD Biosciences, 563994), NK1.1 (1:1000, BD Biosciences, 566502), FOXP3 (1:300, Thermofisher Scientific, 12-5773-82), CD19 (1:300, BD Biosciences, 563333), Rab5 (1:100, CST 46449), EEA1 (1:100 CST 3288), Rab7 (1:100, CST 9367), Rab11 (1:100, CST 5589), Alexa FluorTM 568 (1:500 Invitrogen, A-11077), Goat anti-Mouse IgG (H+L) cross-adsorbed secondary antibody, Alexa FluorTM 488 (1:500 Invitrogen, A-11004), Goat anti-Rabbit IgG (H+L) Cross-Adsorbed Secondary Antibody, Alexa FluorTM 488 (1:500 Invitrogen, A-11008)
Validation	Beclin1 and ATG7 antibodies were validated using corresponding knockout cell lines. For all others no validation was performed other than that provided by manufacturers.

Animals and other research organisms

Policy information about studies involving animals; ARRIVE guidelines recommended for reporting animal research, and Sex and Gender in Research

Laboratory animals	Becn1tm1b(KOMP)Wtsi mice were purchased from the European Conditional Mouse Mutagenesis Program (EUCOMM). Becn1fl/fl mice were generated by breeding Becn1tm1b(KOMP)Wtsi mice onto CAG-FLPe C57BL/6J mice. These, and the Atg7fl/fl (Atg7tm1Tchi) mice were provided by the Kile Laboratory (University of Adelaide, SA, Australia). The Vil1-CreERT2 mice were provided by the Mariadason Laboratory (Olivia Newton-John Cancer Research Institute). The UBC-CreERT2 mice were provided by the Heath Laboratory (Walter and Eliza Hall institute). Male and female mice aged six weeks or older were used.
Wild animals	The study did not involved wild animals.
Reporting on sex	Both male and female mice were used and selection was random.
Field-collected samples	This study did not include field-collected samples.
Ethics oversight	All experiments performed were approved by the La Trobe University animal ethics committees (AECs, AEC18024, AEC18036) in accordance with the Australian code for the care and use of animals for scientific purposes.

Note that full information on the approval of the study protocol must also be provided in the manuscript.

Plants

Seed stocks	N/A
Novel plant genotypes	N/A
Authentication	N/A

Flow Cytometry

Plots

Confirm that:

☒ The axis labels state the marker and fluorochrome used (e.g. CD4-FITC).

☒ The axis scales are clearly visible. Include numbers along axes only for bottom left plot of group (a 'group' is an analysis of identical markers).

☒ All plots are contour plots with outliers or pseudocolor plots.

☒ A numerical value for number of cells or percentage (with statistics) is provided.

Methodology

Sample preparation	For apoptosis assays, organoids were dissociated by incubation for 15 minutes at 37C in TrypLE Express (Gibco) plus pipetting. Cells were stained in the dark for 15 minutes with either FITC Annexin V (BD Pharmingen, 556419) or APC Annexin V (BD Pharmingen, 550475) diluted at 1:20, and Propidium iodide solution (Sigma-Aldrich, P4864) diluted at 1:200 in 1x Annexin V Binding Buffer (BD Pharmingen, 556454). Stained cells were diluted 4x in 1x Annexin V Binding Buffer and analysed by flow cytometry.

Intraepithelial lymphocytes were isolated from the small intestines and colons of mice as previously described. Briefly, excess fat was removed from the colon and small intestines. Peyer's patches were also removed from the small intestines. The tissues were kept moist by frequent saturation with ice-cold wash buffer (dPBS + 2 % (w/v) fetal calf serum). Intestines were cut longitudinally through the lumen, and fecal matter washed away by lightly swirling in a petri dish containing ice-cold wash buffer. Intestines were then cut into 0.5 cm fragments and placed in dissociation solution (wash buffer + 5 mM EDTA) and incubated for 30 minutes at 37°C with gentle shaking. The cell suspension was then vortexed and filtered using a 70 µm cell strainer, transferred to a new 50 mL tube and centrifuged at 1700 rpm for 7 minutes. The cell pellets were then washed twice with wash buffer. Cells were then resuspended in discontinuous 40 % / 80 % Percoll® (GE Healthcare, 17089101) gradient and centrifuged at 900 x g for 20 minutes at room temperature. The interface was collected and washed twice with wash buffer and the final pellet containing intraepithelial lymphocytes stained. For flow cytometry analysis, isolated IELs from above were incubated with Live/Dead marker (1:500, BD Biosciences, 566332), anti-CD45.2 (1:200, BD Biosciences, 741957), CD3ε (1:200, BD Biosciences, 564378), CD4 (1:1000, BD Biosciences, 741461), CD8α (1:1000, BD Biosciences, 562283), CD8β (1:1000, BD Biosciences, 741127), TCRβ (1:300, ThermoFisher Scientific, 47-5961-82), TCRγδ (1:300, BD Biosciences, 563994), NK1.1 (1:1000, BD Biosciences, 566502), FOXP3 (1:300, Thermofisher Scientific, 12-5773-82), and CD19 (1:300, BD Biosciences, 563333) for total IEL analysis. For intracellular staining, cells were fixed and permeabilised using the Foxp3/Transcription factor staining buffer kit (Invitrogen, 00-5523-00) according to the manufacturer's instructions, followed by incubation with intracellular markers. Finally, cells were resuspended in FACS wash buffer containing CountBright Absolute Counting Beads (Molecular Probes, C36950) and stained cell acquisition was performed.

Whole-mount intestinal organoid staining protocol was adapted from Dekkers et al . Briefly, organoids were removed from matrix by incubation with ice-cold Gentle Cell Dissociation Reagent (STEMCELL Technologies, 100-0485) with gentle rocking at 4°C for 60 minutes. Organoids were then fixed in 4 % (w/v) paraformaldehyde solution (ProSciTech, Catalog no. C004) and blocked with Organoid Wash Buffer (DPBS (GibcoTM, 14190144) + 0.1% (w/v) Triton X-100 (Merck, X100) + 0.2 % (w/v) Bovine Serum Albumin (Merck, A3059)), followed by overnight incubation at 4°C with gentle rocking with the stated primary antibodies.

Instrument

Flow cytometry performed using the BD FACSCanto II (Becton Dickinson and Company, BD Biosciences, San Jose, CA, USA) and BD FACSymphonyTM A3 Cell Analyzer (Becton Dickinson and Company, BD Biosciences, San Jose, CA, USA)

Software

FLOWJO v10.5.3

Cell population abundance

As only population analysis was undertaken, no sorting for specific populations was undertaken.

Gating strategy

For cell death assays, cells were initially gated on FSC-A/SSC-A then further gated for live/dead populations using appropriate filter sets for FITC or APC (Annexin V) and propidium iodide. % Live cells were calculated as those that were negative for Annexin V and propidium iodide. For IEL analysis, cells were initially gated using FSC-A against SSC-A to obtain lymphocyte populations, and doublets were excluded by gating using FSC-A against FSC-H. Cells were further gated using CD45.2 and viability (Live/dead) marker to obtain live haematopoietic cell populations. The T-cell population was gated from the live haematopoietic cell population using CD3ε. The regulatory T-cell population was gated from these using CD4 and FoxP3 markers, whereas the γδ and αβ T-cell subpopulations were further gated using TCRγδ and TCRβ markers, respectively. The γδ and αβ T-cell subpopulations were also further gated with CD4, CD8α and CD8β markers to obtain CD8αα+γδ+ T cells, CD8αβ+γδ+ T cells, and CD8αβ-γδ+ T cells, as well as CD4+αβ+ T cells, CD8αα+αβ+ T cells, CD8αβ+αβ+ T cells, and CD8αβ-αβ+ T cells populations. The B-cell population was obtained by gating populations that were CD3ε-negative and CD19-positive. The Natural killer cell population was obtained by gating cells that were negative for CD3ε and positive for the NK1.1 marker.

☒ Tick this box to confirm that a figure exemplifying the gating strategy is provided in the Supplementary Information.
